# Apolipoprotein E epsilon-4 polymorphism is associated with younger age at referral to a lipidology clinic and a poorer response to lipid-lowering therapy

**DOI:** 10.1186/1476-511X-10-48

**Published:** 2011-03-30

**Authors:** Rui Baptista, Marta Rebelo, Joana Decq-Mota, Patrícia Dias, Pedro Monteiro, Luís A Providência, José M Silva

**Affiliations:** 1 Department of Cardiology, Coimbra Hospital and Medical School, Coimbra, Portugal; 2 Department of Internal Medicine, Coimbra Hospital and Medical School, Coimbra, Portugal

## Abstract

**Background:**

The risk of coronary heart disease (CHD) is related to environmental factors and genetic variants. Apolipoprotein E (apoE) polymorphisms are heritable determinants of total and low-density lipoprotein cholesterol, with some authors suggesting an association between the ε4 allele and CHD. We investigated the relationship between apoE genotype and age at referral to a specialized lipid clinic by the primary care physician and whether the benefits of treatment with statin differed between genotypes.

**Methods:**

We assessed individual apoE genotypes and lipid blood profile in a total of 463 patients followed at a specialized lipid clinic due to dyslipidemia, with a 3-year median follow-up time. The primary care physician at the time of the referral had no access to the apoE genotyping results. Carriers of apoE ε4/ε2 genotype were excluded.

**Results:**

The frequencies of ε2, ε3 and ε4 alleles were 7.8, 78.9 and 13.3%, respectively. There were no significant differences between genders. Although with similar lipid profiles and antidyslipidemic drug usage at baseline, ε4-carriers were referred to the clinic at a younger age (44.2 ± 14.7 years) compared with non-ε4 carriers (50.6 ± 13.8 years) (p < 0.001), with a substantially younger age of referral for homozygous E4/4 and for all genotypes with at least one copy of the ε4 allele (p < 0.001 for trend). Although both ε4 and non-ε4 carriers achieved significant reductions in total cholesterol during follow-up (p < 0.001 vs. baseline), the mean relative decrease in total cholesterol levels was higher in non-ε4 carriers (-19.9 ± 2.3%) compared with ε4 carriers (-11.8 ± 2.3%), p = 0.003.

**Conclusion:**

Our findings support the concept that there is a reduced response to anti-dyslipidemic treatment in ε4 carriers; this can be a contributing factor for the earlier referral of these patients to our specialized lipid clinic and reinforces the usefulness of apoE genotyping in predicting patients response to lipid lowering therapies.

## Background

Coronary heart disease (CHD) is the main cause of mortality in developed countries. The risk of CHD may be influenced by environmental factors and genetic mutations in a number of genes controlling blood lipids and other risk factors. One of the most studied genes is the one coding for apolipoprotein E (apoE), located in chromosome 9 [1]. ApoE is a serum glycoprotein that plays a critical role in lipid metabolism[2]. It serves as a ligand for cell-surface receptor uptake of chylomicrons and very low-density lipoproteins (VLDL) on the liver [3] and controls intestinal cholesterol absorption[4].

A common polymorphism in the *APOE *gene (rs429358, rs7412) results in three isoforms called apoE2, apoE3 and apoE4, which are coded by three codominant alleles (designated as ε2, ε3 and ε4), giving rise to six different genotypes [5]. The impact of apoE on plasma lipids is well known and may be partly related with increased CHD risk, with increasing plasma total cholesterol (TC) and low density lipoprotein (LDL) cholesterol in the presence of the ε4 allele [6-8]. However, the effect on HDL, apolipoprotein (apo) A or lipoprotein (Lp) (a) is less clear[9].

The results of epidemiologic studies examining the association between apoE genotypes and cardiovascular outcomes are inconsistent. Earlier data pointed to a clear gradient of risk conferred by the ε4 allele, with ε4-carriers particularly prone to develop disseminated coronary lesions, to be submitted to coronary revascularization procedures and to die from CHD [10-13]. However, these results have been questioned by Ward *et al*., that found no association between apoE genotypes and CHD in a recent population study[14].

Based on this, the purpose of our investigation was (1) to determine the apoE genotype distribution and its association with plasma lipid traits in a population referred to a specialized lipid outpatient clinic, (2) to investigate whether the ε4 allele influences the age of referral to the clinic, and if this was the case, (3) to assess if patients with the ε4 allele were poorer responders to lipid-lowering therapeutic interventions.

## Methods

### Study design and participants

We conducted a retrospective observational study based on the analysis of the clinical records of 691 patients consecutively admitted and followed in a tertiary hospital specialized dyslipidemia outpatient clinic between January 1994 and October 2007. All patients were referred to consultation by their primary care physician or by other specialist within the hospital due to markedly abnormal, difficult to control lipid profile or due to suspected familial dyslipidemia. All patients were followed in either six-monthly or annual outpatient consultations, as decided appropriate by the attending physician, with regular assessment of laboratory and clinical parameters. All patients were treated to target the proposed LDL level recommended by the ATP III guidelines[15], by the use of dietary and pharmacological strategies. No formal recommendations were given regarding which type of drugs should be used for reaching the lipidic target. Apolipoprotein E genotypes were not taken in account when making therapeutic decisions regarding lipid-lowering drugs, as genotyping was performed solely with an investigational purpose. We conducted a 3-year follow-up analysis of blood lipids, namely TC, LDL, HDL and triglycerides. The study was approved by the local institutional board and all patients gave informed consent.

### Risk factors assessment

Baseline, demographic and clinical variables are collected, including age at referral, gender, prior history of CHD, diabetes *mellitus*, smoking status, alcohol consumption, use of a salty diet and practice of exercise. Diabetes was defined as fasting glucose ≥ 126 mg/dL or use of hypoglycemic drugs and actual smoker if smoking one or more cigarettes per day in the last year. Alcohol consumption was positive in the patient consumed more than two units of alcoholic beverages per day. Use of a salty diet was considered if the patient added table salt to food. Regular exercise was defined by the performance of three or more periods of moderate exercise per week. On the first consultation, several baseline laboratory variables were obtained and a complete lipid profile was assayed by standard techniques in 12-h fasting blood samples, including TC, HDL, LDL, triglycerides, apoA and apoB and Lp(a) levels. Peripheral blood samples were obtained from each patient and dispatched immediately to analysis in our hospital central laboratory at 4°C before processing.

### DNA extraction and apoE genotyping

DNA was extracted from whole-blood specimens according to standard procedures. Genomic DNA from these samples was analyzed for apoE polymorphisms (rs7412 and rs429358) using polymerase chain reaction and reverse hybridization; apoE concentrations were measured by nephelometry. Importantly, at the time of referral, no referring physician was aware of the apoE genotype of the patient, as these tests were only requested after this first medical contact in the hospital.

### Statistical analysis

Allele frequencies were determined using the gene counting method. Hardy-Weinberg equilibrium for the distribution of the genotype was performed. Continuous variables were expressed as mean ± SD. Median and interquartile range were used if the distribution was not normal, assessed by the use of the Kolmogorov-Smirnov test. The Student unpaired t-test for normal variables and the Mann-Whitney U test for non-normal variables were used for comparisons among groups. Paired-samples T-test was used for comparison between baseline and follow-up assessments. Categorical variables were presented as percentages, and were compared using χ2 or Fisher's exact test.

The number of patients in some individual genotype groups was too small to support group comparisons; therefore, and in a similar way to several other reports, we next compared patients with one or more copies of the ε4 allele (ε4-carriers) to those without (non-ε4 carriers). As in many other studies of this nature, eight subjects with the E4/2 genotype were excluded from the subsequent analyses since they could not be simply classified into any single allele group[14], because of the putative opposite effects of these two alleles in lipid levels. Plasma Lp(a) and triglycerides values were logarithmically transformed before all statistical analyses to improve the normality of the distributions. All statistical analyses were performed using SPSS 13.0 (Chicago, Illinois) with the level of significance set at p < 0.05.

## Results

Of the 691 patients who were studied in the outpatient lipid consult, 228 were not genotyped, leaving a final sample with complete data for 463 patients, all Caucasian of Southern European ancestry. The observed distribution of apoE genotypes among patients, separately by man and women, is presented in Table 1. The frequencies of ε2, ε3 and ε4 alleles were 7.8, 78.9 and 13.3%, respectively. Overall, 112 of 463 patients (24.2%) were carriers of the ε4 allele and 351 (75.8%) were non-ε4 carriers. The distribution of the apoE alleles was not in Hardy-Weinberg equilibrium. No significant differences were found among gender regarding the distribution of the alleles.

**Table 1:** Frequency of apoE genotype and apoE allele by gender

	Total	Male	Female
*N*	463	272	192
Genotype			
E2/2	11 (2.4%)	7 (2.6%)	4 (2.1%)
E3/2	42 (9.1%)	26 (9.6%)	16 (8.3%)
E4/2	8 (1.7%)	4 (1.5%)	4 (2.1%)
E3/3	298 (64.2%)	172 (63.2%)	126 (65.6%)
E4/3	93 (20.0%)	56 (20.6%)	37 (19.3%)
E4/4	11 (2.6%)	6 (2.2%)	5 (2.7%)
Allele			
ε2	7.8%	8.1%	7.3%
ε3	78.9%	78.6%	79.4%
ε4	13.3%	13.3%	13.3%

The apoE ε4-carriers and non-ε4 carriers groups were compared regarding demographic, clinical and laboratory variables (Table 2 and 3). The studied population had a small percentage of patients with prior CHD (4.2%) but we found a significant proportion of diabetic patients (24.9%). At baseline, no differences were found among patients with and without the ε4 allele with regards to gender, smoking habits, alcohol consumption, exercise or use of a salty diet. Diabetes *mellitus *was less prevalent in patients with the ε4 allele, but not reaching statistical significance (20.5 vs. 26.5%, p = 0.286). No differences were found regarding aspirine, angiotensin receptor blockers, beta-blockers or calcium channel antagonists usage among groups. Angiotensin-converting enzyme inhibitors (ACEi) were used more frequently in non-ε4 carriers; these patients also presented with higher blood pressure, although the average values were in the normal range (135.6 ± 21.0 vs. 127.9 ± 17.6 mmHg, p = 0.002). Regarding prior antidyslipidemic drug usage, including statins and fibrates, no significant differences were found between ε4 carriers and non-ε4 carriers.

**Table 2:** Baseline characteristics of the 455 patients with different apoE alleles

	Total	Non-ε4 carriers	ε4 carriers	p-value
Age, years	49.2 ± 14.2	50.6 ± 13.8	44.2 ± 14.7	<0.001
Men [n (%)]	267 (58.7%)	156 (58.4%)	159 (59.6%)	0.826
BMI, kg.m^-2^	28.1 ± 4.7	28.1 ± 4.8	28.1 ± 4.1	0.896
SBP, mmHg	133.8 ± 20.5	135.6 ± 21	127.9 ± 17.6	0.002
DBP, mmHg	83.5 ± 11.7	84.2 ± 11.5	80.9 ± 12.1	0.016
Heart rate, bpm	73.4 ± 12.3	73.5 ± 11.9	73.3 ± 13.8	0.915
TC, mg.dℓ^-1^	275.9 ± 77.8	277.8 ± 81.3	269.4 ± 64.4	0.338
LDL, mg.dℓ^-1^	157.7 ± 57.3	157.6 ± 58.5	158.0 ± 53.2	0.956
HDL, mg.dℓ^-1^	49.6 ± 23.3	49.9 ± 24.9	48.6 ± 17.0	0.618
Triglycerides, mg.dℓ^-1^	210 (125-389)	218 (127-381)	177 (108-437)	0.535^1^
Lp(a) (mg.dℓ^-1^)	15 (6 - 37)	15 (6 - 37)	14 (6 - 36)	0.815^1^
ApoE (mg.dℓ^-1^)	6.8 ± 5.9	7.3 ± 6.4	5.3 ± 3.0	0.008
ApoB (mg.dℓ^-1^)	143.3 ± 46.8	145.7 ± 49.3	135.5 ± 36.5	0.062
ApoA (mg.dℓ^-1^)	152.4 ± 32.9	154 ± 32.4	146.8 ± 33.9	0.062
ApoB/ApoA	0.98 ± 0.36	1.0 ± 0.4	1.0 ± 0.3	0.593
Creatinine, mg.dℓ^-1^	0.96 ± 0.49	1.0 ± 0.5	0.9 ± 0.3	0.495
CK (U.ℓ^-1^)	117.1 ± 123.3	114.1 ± 83.9	127.3 ± 206.2	0.400

^1 ^Comparisons for natural log-transformed variables. Values are median (interquartile range). BMI: body mass index. SBP: systolic blood pressure; DBP: diastolic blood pressure; TC: total cholesterol; LDL: low-density lipoprotein; HDL: high density lipoprotein; Lp(a): lipoprotein (a); Apo: apolipoprotein; CK: creatine kinase.

**Table 3:** Baseline prior history and medication of the 455 patients with different apoE alleles

	Total	Non-ε4 carriers	ε4 carriers	Odds-ratio (95% CI)	p-value
Prior history					
Diabetes mellitus	24.9%	26.3%	20.5%	0.723 (0.398-1.313)	0.285
Prior CHD	4.2%	4.9%	2.4%	0.478 (0.105 - 2.179)	0.330
Current smoker	21.2%	22.4%	17.1%	0.712 (0.375 - 1.353)	0.298
Alcohol consumption	52.8%	55.1%	45.1%	0.670 (0.408 - 1.101)	0.112
Regular exercise	10.6%	11.2%	8.4%	0.728 (0.308 - 1.720)	0.467
Use of a salty diet	57.3%	58.2%	54.2%	0.851 (0.519 - 1.395)	0.522
Prior medication					
Aspirin	41.8%	44.5%	32.9%	0.612 (0.357 - 1.048)	0.072
Beta-blockers	22.0%	23.7%	16.7%	0.646 (0.325 - 1.284)	0.210
ACEi	33.3%	36.4%	23.0%	0.522 (0.287 - 0.950)	0.032
ARB	21.3%	21.9%	19.2%	0.817 (0.415 - 1.607)	0.614
Calcium antagonist	19.2%	19.9%	16.9%	0.817 (0.415 - 1.607)	0.557
Hypolipidemic drug*	81.6%	83.1%	76.5%	0.663 (0.344 - 1.277)	0.217

ACEi: angiotensin converting enzime inhibitor: ARB: angiotensin receptor blocker *Statin or fibrate.

**Figure 1:**
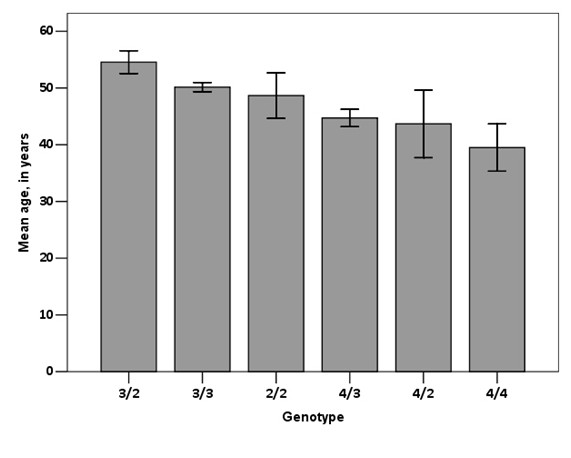
**Mean age at referral for the specialized lipid clinic, by genotype**. Bars are one standard error. P < 0.001 for trend.

However, regarding age at referral to the lipid clinic, ε4-carriers were referred to hospital by their primary care physicians at significant younger ages than non-ε4 carriers (44.2 ± 14.7 vs. 50.6 ± 13.8 years, p < 0.001). We also separated mean age at referral by genotype (Figure 1). The comparison of age at referral by the six apoE genotypes showed a significant effect, with a substantially younger age of referral for homozygous E4/4 and for all genotypes with at least one copy of the ε4 allele (p < 0.001 for trend). Interestingly, at baseline, ε4-carriers had similar lipid profiles compared with non-ε4 carriers, independently of the fraction analyzed (TC, LDL, HDL, triglycerides, Lp(a), ApoA or ApoB). The only significant difference found in lipid profile was in apoE concentration with ε4-carriers having lower values as expected (5.3 ± 3.0 vs. 7.3 ± 6.4 mg.dℓ^-1^, p = 0.008). Other biochemical variables, as creatinine or creatine phosphokinase, as a marker of statin side effects, were similar among groups and with average values within the expected range.

### Outpatient clinical follow-up

**Figure 2:**
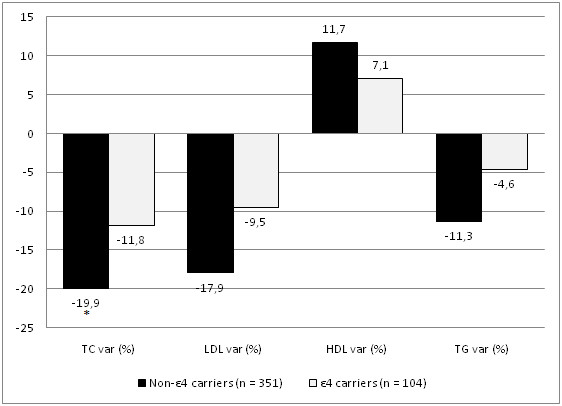
**Three-year follow-up variation in lipid levels, by ε4-carrier status**. TC: total cholesterol; LDL: low-density lipoprotein; HDL: high density lipoprotein; TG: triglyceride; var: variation. * P < 0.05.

All patients were followed in the specialized lipid clinic for a median period of 3 years (interquartile range, 1 to 6). During this period, a strategy was implemented in order to achieve the recommended ATP III goal levels of cholesterol in all patients. The variation in lipid profile is shown on Table 4. After the follow-up period, 57% of patients achieved a LDL value inferior to 130 mg.dℓ^-1 ^and 32% an LDL inferior to 100 mg.dℓ^-1^. The mean variation in TC was -18.2%, whereas HDL went up 11.1% and triglycerides were reduced by -10.4% (all p < 0.001 vs. baseline values). Significant differences were found among ε4-carriers and non- ε4 carriers regarding the effectiveness of the therapeutic intervention. The proportion of patients reaching the LDL target level was lower in the ε4-carriers and the absolute difference between TC at baseline and after therapeutic intervention was 54.91 ± 67.0 mg.dℓ^-1 ^in the ε4 carriers, compared with 78.8 ± 66.9 mg.dℓ^-1 ^in the ε4-non carriers (p < 0.05). This correlates with an inferior relative reduction in TC within ε4-carriers than in ε4-non carriers (-11.8 ± 2.3% vs. -19.9 ± 2.3%, p = 0.003). The same was true for the other lipid subfractions, but without reaching statistical significance (Figure 2).

**Table 4:** Follow-up lipid profile of patients

	Total	Non-ε4 carriers	ε4 carriers	p-value
LDL < 130 mg.dℓ^-1 ^(%)	57.2	61.4	43.2	0.002
LDL < 100 mg.dℓ^-1 ^(%)	32.4	34.9	23.9	0.052
TC variation (%)	-18.2 ± 2.3	-19.9 ± 2.3	-11.8 ± 2.3	0.003
LDL variation (%)	-16.0 ± 4.7	-17.9 ± 4.9	-9.5 ± 4.3	0.068^1^
HDL variation (%)	+11.1 ± 4.3	+11.7 ± 4.7	+7.1 ± 2.6	0.379^1^
Triglyceride variation (%)	-10.4 ± 8.5	-11.3 ± 8.7	-4.6 ± 7.8	0.508^1^

^1 ^Mann-Whintey test. TC: total cholesterol; LDL: low-density lipoprotein; HDL: high density lipoprotein; TG: triglyceride.

## Discussion

To our knowledge this is the first study to examine the association between apoE genotypes and age at referral to a specialized lipid clinic. We demonstrate that patients carrying one or more copies of the ε4 allele are referred at significantly younger ages to the lipid clinic and after a mean follow-up of three years these patients have a poorer response to treatment.

We observed overall that the ε3 allele was the most frequent, followed by the ε4 allele and ε2 allele. This finding is ubiquitous for human populations [9]. In Europe, the frequency of apoE genotypes demonstrates a North-South gradient, with a progressive decline in the ε4 allele frequency from Northern (14-19%) to Southern Europe (7-12%) [16]. In our population the frequency of the ε4 allele was 13.3%, a value that is closer to the one found in populations from Northern Europe and in clear excess of the expected frequency of the ε4 allele in the Portuguese healthy population (9.0%), one of the lowest reported in Europe [7]. This disequilibrium might be explained by the referred nature of our population, although that fact is not obvious by the lipid concentrations, as there are no significant differences among groups at baseline. Regarding the E2/2 genotype, there is also a higher frequency (2.2%) than expected for the Portuguese population (0.3%)[7], probably due to the inclusion of some cases of hyperlipoproteinemia type III[17].

ApoE ε4 allele has been linked to higher total and LDL cholesterol plasma levels in several studies[7]. However, this was not the case in our population, where we found no significant association between ε4-carrier status and baseline TC and LDL. The associations between the apoE polymorphism and plasma concentrations of HDL, apoA, Lp(a) and triglycerides have been more inconsistent in the literature, with results that range from no association[18,19] to associations that vary by sex [20]. We found no association between those lipid traits and ε4-carrier status in our population. We also found, as expected, that ε4-carriers had lower plasma concentrations of apoE, compared with ε4-non carriers. It has been suggested that high apoE plasma levels have prognostic impact, independent of apoE genotypes, lipid values and other cardiovascular risk factors [7,21]. Further research is needed to clarify to role of apoE concentration in the assessment of global cardiovascular risk.

As reported by Chiodoni *et al.*[22], we found a non-significant trend of a lower prevalence of diabetic patients in ε4-carriers compared with the non-ε4 carriers, this disease being highly prevalent in our cohort (21.1%). This can be due to the younger age of presentation of the ε4-carriers or to a distinctive effect of the ε4 allele on carbohydrate metabolism that remains to be elucidated.

Although no differences were found in lipid profiles among ε4-carriers and non-carriers, the former were consistently referred at younger ages to the lipid clinic, with a clear and significant gradient to patients with more copies of the ε4 allele being referred at proportionally younger ages. What alerted the primary care physicians to refer such patients at younger ages?

Several reports studied the interaction between age, apoE and target organ disease. Newman *et al. *demonstrates a significant trend for early coronary revascularization surgery for patients with higher number of copies of the ε4 allele, compared with ε3 and ε2 carriers[12]. Other studies have also indicated a substantial (16-fold) increase in the prevalence of the ε4 allele in patients referred for coronary angioplasty[23] and its predictive power regarding coronary vessel restenosis [24]. In our population, although not having a higher incidence of prior CHD, ε4-carriers could have presented with more intense abnormalities in lipid profile than expected at younger ages to their primary care physicians, prompting the referral to the hospital.

Other factor that may have been implicated in early referral was the level of response to lipid-lowering therapy. During follow-up, the TC of our patients was lowered in average 18%, the LDL-C 16% and TG 10% and HDL was improved on average 11%. These results compare with those from the GISSI-Prevenzione (GISSI-P) study, also conducted in a Southern European population and that obtained at a mean follow-up time of 24 months similar reductions in the lipid profile with the use of pravastatin (20 to 40 mg) [25]. We report a lower variation in TC in ε4-carriers compared to non-ε4 carriers, with both groups under a similar hypolipidemic strategy (-19.9 ± 2.3 vs. -11.8 ± 2.3%, p = 0.003). In fact, although departing from slightly lower TC levels, ε4-carriers reached the end the follow-up period with higher TC levels than non-ε4 carriers and with a higher proportion of patients with LDL over 130 mg.dℓ^-1^.

The effect of the apoE genotype on antidyslipidemic drugs' efficacy, namely statins, has been thoroughly studied in the last years[26]. Although several efforts have been made to identify genes that might be involved in statin response, a recent analysis for the Treating to New Targets (TNT) study concluded that, after analyzing almost 300,000 putative sites, only polymorphisms in the apoE gene were found to influence statin response significantly, highlighting the pivotal role of this gene on lipid metabolism. In this genome-wide study, patients with apoE 4/4 allele had a lower response to atorvastatin treatment than patients with E3/3 allele regarding LDL levels (37.7 vs. 40.3%) [27]. Taken together, the majority of studies indicate that patients with the ε4 allele seem to have the poorest and those with the ε2 allele the strongest response to statins regarding LDL lowering [28-31]; in addition, ε2 carriers may reach the LDL treatment goals more frequently than ε4 carriers [32]. These findings have not been reproduced by other authors [33-35]. In a subanalysis of the GISSI-P study, only carriers of the considered high-risk ε4 allele seemed to benefit from the treatment with statin, regarding mortality [22], corroborating the findings of the 4S subanalysis, where the treatment with simvastatin neutralized the higher risk of mortality of ε4 carriers[33]. This fact should alert physicians to aggressively treat to target lipid goals in these patients.

Our population was selected by primary care physicians for follow-up at a specialized lipid clinic and does not represent a cross-section of the population as a whole; however, based on cardiovascular risk factors, our population reflects the usual adult population seen in a primary care setting. The reported disequilibrium in genotype frequencies compared to those expected in a non-selected healthy population reflects the referral nature of our population. Numerous factors may have guided the decision to refer to lipid clinic and we can only speculate on what those factors may be. However, the fact remains that in the population studied, there was a distinct association between the presence of the apoE ε4 allele and an earlier presentation for tertiary specialized lipid care. We also demonstrate that after a mean follow-up of three years, the impact of therapeutics in the level of TC was significantly lower in ε4-carriers. One may hypothesize that, although having similar lipid levels at baseline compared with non-ε4 carriers, the combination of a more severe lipid profile at younger ages with a poorer response to an already initiated standard hypolipidemic therapy may all had a role in the decision of the primary care physician to refer these patients. We must highlight that primary care physicians were blinded to apoE genotypes, as genotyping was performed only at the hospital.

The study has some limitations. The inclusion of participants receiving medication for CHD, diabetes, hypertension or hypercholesterolemia can alter physiologic lipid levels and confound the lipid associations. However, no differences were found regarding the frequency of usage of these medications in primary care between groups, except for ACEi. Moreover, as the primary care physicians were not aware of the genotypes, the referral was made in an entirely blinded fashion. The inexistence of a defined protocol for statin use during follow-up time in the specialized lipid consultation is compensated by the fact that no differences were found on lipid-lowering therapies management on both groups.

## Conclusions

In conclusion, we have demonstrated that the presence of the apoE ε4 allele is associated with referral at younger ages to a specialized lipid clinic, although no differences were found regarding baseline lipid profile that could account for such earlier referrals. Moreover, these patients had a poorer response to the antidyslipidemic treatment instituted during the follow up period. Intensive therapeutical strategies must be followed to achieve the recommended target goals of lipid levels, particularly in high-risk patients as ε4-carriers. Our findings suggest that pharmacogenomics could be considered for individualized tailoring of global cardiovascular risk assessment strategies.

## Competing interests

The authors declare that they have no competing interests.

## Authors' contributions

RB conceived the study, participated in the design of the study, acquired data and performed the statistical analysis. MR participated in the design of the study and acquired data. JDM participated in the design of the study and acquired data. PD participated in the design of the study and its coordination. PM revised the manuscript for important intellectual content. LAP revised the manuscript for important intellectual content. JMS conceived the study, participated in the design of the study and revised the manuscript for important intellectual content.

All authors read and approved the final manuscript.

## References

[B1] Davignon J Gregg RE Sing CF Apolipoprotein E polymorphism and atherosclerosis Arteriosclerosis 1988 8 1 21 3277611 10.1161/01.atv.8.1.1

[B2] Kolovou GD Anagnostopoulou KK Apolipoprotein E polymorphism, age and coronary heart disease Ageing Res Rev 2007 6 94 108 10.1016/j.arr.2006.11.001 17224309

[B3] Anoop S Misra A Meena K Luthra K Apolipoprotein E polymorphism in cerebrovascular & coronary heart diseases Indian J Med Res 2010 132 363 378 20966513

[B4] Kesaniemi YA Ehnholm C Miettinen TA Intestinal cholesterol absorption efficiency in man is related to apoprotein E phenotype J Clin Invest 1987 80 578 581 10.1172/JCI113107 3611358 PMC442272

[B5] Mahley RW Rall SC Jr Apolipoprotein E: far more than a lipid transport protein Annu Rev Genomics Hum Genet 2000 1 507 537 10.1146/annurev.genom.1.1.507 11701639

[B6] Lehtinen S Lehtimaki T Sisto T Salenius JP Nikkila M Jokela H Koivula T Ebeling F Ehnholm C Apolipoprotein E polymorphism, serum lipids, myocardial infarction and severity of angiographically verified coronary artery disease in men and women Atherosclerosis 1995 114 83 91 10.1016/0021-9150(94)05469-Y 7605379

[B7] Haddy N De Bacquer D Chemaly MM Maurice M Ehnholm C Evans A Sans S Do Carmo Martins M De Backer G Siest G Visvikis S The importance of plasma apolipoprotein E concentration in addition to its common polymorphism on inter-individual variation in lipid levels: results from Apo Europe Eur J Hum Genet 2002 10 841 850 10.1038/sj.ejhg.5200864 12461692

[B8] Alvim RO Freitas SR Ferreira NE Santos PC Cunha RS Mill JG Krieger JE Pereira AC APOE polymorphism is associated with lipid profile, but not with arterial stiffness in the general population Lipids Health Dis 2010 9 128 10.1186/1476-511X-9-128 21059196 PMC2992057

[B9] Burman D Mente A Hegele RA Islam S Yusuf S Anand SS Relationship of the ApoE polymorphism to plasma lipid traits among South Asians, Chinese, and Europeans living in Canada Atherosclerosis 2009 203 192 200 10.1016/j.atherosclerosis.2008.06.007 18656198

[B10] Eichner JE Kuller LH Orchard TJ Grandits GA McCallum LM Ferrell RE Neaton JD Relation of apolipoprotein E phenotype to myocardial infarction and mortality from coronary artery disease Am J Cardiol 1993 71 160 165 10.1016/0002-9149(93)90732-R 8421977

[B11] Stengard JH Zerba KE Pekkanen J Ehnholm C Nissinen A Sing CF Apolipoprotein E polymorphism predicts death from coronary heart disease in a longitudinal study of elderly Finnish men Circulation 1995 91 265 269 7805227 10.1161/01.cir.91.2.265

[B12] Newman MF Laskowitz DT White WD Kirchner JL Grocott HP Stafford-Smith M Sketch MH Jones RH Reves JG Saunders AM Apolipoprotein E polymorphisms and age at first coronary artery bypass graft Anesth Analg 2001 92 824 829 10.1097/00000539-200104000-00006 11273909

[B13] Bennet AM Di Angelantonio E Ye Z Wensley F Dahlin A Ahlbom A Keavney B Collins R Wiman B de Faire U Danesh J Association of apolipoprotein E genotypes with lipid levels and coronary risk JAMA 2007 298 1300 1311 10.1001/jama.298.11.1300 17878422

[B14] Ward H Mitrou PN Bowman R Luben R Wareham NJ Khaw KT Bingham S APOE genotype, lipids, and coronary heart disease risk: a prospective population study Arch Intern Med 2009 169 1424 1429 10.1001/archinternmed.2009.234 19667307

[B15] Third Report of the National Cholesterol Education Program (NCEP) Expert Panel on Detection, Evaluation, and Treatment of High Blood Cholesterol in Adults (Adult Treatment Panel III) final report Circulation 2002 106 3143 3421 12485966

[B16] Eichner JE Dunn ST Perveen G Thompson DM Stewart KE Stroehla BC Apolipoprotein E polymorphism and cardiovascular disease: a HuGE review Am J Epidemiol 2002 155 487 495 10.1093/aje/155.6.487 11882522

[B17] Smelt AH de Beer F Apolipoprotein E and familial dysbetalipoproteinemia: clinical, biochemical, and genetic aspects Semin Vasc Med 2004 4 249 257 10.1055/s-2004-861492 15630634

[B18] Anuurad E Lu G Rubin J Pearson TA Berglund L ApoE genotype affects allele-specific apo[a] levels for large apo[a] sizes in African Americans: the Harlem-Basset Study J Lipid Res 2007 48 693 698 10.1194/jlr.M600431-JLR200 17172560

[B19] de Knijff P Kaptein A Boomsma D Princen HM Frants RR Havekes LM Apolipoprotein E polymorphism affects plasma levels of lipoprotein(a) Atherosclerosis 1991 90 169 174 10.1016/0021-9150(91)90111-F 1836947

[B20] Frikke-Schmidt R Nordestgaard BG Agerholm-Larsen B Schnohr P Tybjaerg-Hansen A Context-dependent and invariant associations between lipids, lipoproteins, and apolipoproteins and apolipoprotein E genotype J Lipid Res 2000 41 1812 1822 11060351

[B21] Mooijaart SP Berbee JF van Heemst D Havekes LM de Craen AJ Slagboom PE Rensen PC Westendorp RG ApoE plasma levels and risk of cardiovascular mortality in old age PLoS Med 2006 3 e176 10.1371/journal.pmed.0030176 16671834 PMC1457005

[B22] Chiodini BD Franzosi MG Barlera S Signorini S Lewis CM D'Orazio A Mocarelli P Nicolis E Marchioli R Tognoni G Apolipoprotein E polymorphisms influence effect of pravastatin on survival after myocardial infarction in a Mediterranean population: the GISSI-Prevenzione study Eur Heart J 2007 28 1977 1983 10.1093/eurheartj/ehm196 17567623

[B23] van Bockxmeer FM Mamotte CD Apolipoprotein epsilon 4 homozygosity in young men with coronary heart disease Lancet 1992 340 879 880 10.1016/0140-6736(92)93288-X 1357300

[B24] van Bockxmeer FM Mamotte CD Gibbons FR Taylor RR Apolipoprotein epsilon 4 homozygosity--a determinant of restenosis after coronary angioplasty Atherosclerosis 1994 110 195 202 10.1016/0021-9150(94)90204-6 7848369

[B25] Results of the low-dose (20 mg) pravastatin GISSI Prevenzione trial in 4271 patients with recent myocardial infarction: do stopped trials contribute to overall knowledge? GISSI Prevenzione Investigators (Gruppo Italiano per lo Studio della Sopravvivenza nell'Infarto Miocardico) Ital Heart J 2000 1 810 820 11302109

[B26] Nieminen T Kahonen M Viiri LE Gronroos P Lehtimaki T Pharmacogenetics of apolipoprotein E gene during lipid-lowering therapy: lipid levels and prevention of coronary heart disease Pharmacogenomics 2008 9 1475 1486 10.2217/14622416.9.10.1475 18855536

[B27] Thompson JF Hyde CL Wood LS Paciga SA Hinds DA Cox DR Hovingh GK Kastelein JJ Comprehensive whole-genome and candidate gene analysis for response to statin therapy in the Treating to New Targets (TNT) cohort Circ Cardiovasc Genet 2009 2 173 181 10.1161/CIRCGENETICS.108.818062 20031582

[B28] Ballantyne CM Herd JA Stein EA Ferlic LL Dunn JK Gotto AM Jr Marian AJ Apolipoprotein E genotypes and response of plasma lipids and progression-regression of coronary atherosclerosis to lipid-lowering drug therapy J Am Coll Cardiol 2000 36 1572 1578 10.1016/S0735-1097(00)00918-9 11079660

[B29] Nestel P Simons L Barter P Clifton P Colquhoun D Hamilton-Craig I Sikaris K Sullivan D A comparative study of the efficacy of simvastatin and gemfibrozil in combined hyperlipoproteinemia: prediction of response by baseline lipids, apo E genotype, lipoprotein(a) and insulin Atherosclerosis 1997 129 231 239 10.1016/S0021-9150(96)06031-5 9105566

[B30] Ojala JP Helve E Ehnholm C Aalto-Setala K Kontula KK Tikkanen MJ Effect of apolipoprotein E polymorphism and XbaI polymorphism of apolipoprotein B on response to lovastatin treatment in familial and non-familial hypercholesterolaemia J Intern Med 1991 230 397 405 10.1111/j.1365-2796.1991.tb00464.x 1940775

[B31] Ordovas JM Lopez-Miranda J Perez-Jimenez F Rodriguez C Park JS Cole T Schaefer EJ Effect of apolipoprotein E and A-IV phenotypes on the low density lipoprotein response to HMG CoA reductase inhibitor therapy Atherosclerosis 1995 113 157 166 10.1016/0021-9150(94)05439-P 7605354

[B32] Donnelly LA Palmer CN Whitley AL Lang CC Doney AS Morris AD Donnan PT Apolipoprotein E genotypes are associated with lipid-lowering responses to statin treatment in diabetes: a Go-DARTS study Pharmacogenet Genomics 2008 18 279 287 10.1097/FPC.0b013e3282f60aad 18334912

[B33] Gerdes LU Gerdes C Kervinen K Savolainen M Klausen IC Hansen PS Kesaniemi YA Faergeman O The apolipoprotein epsilon4 allele determines prognosis and the effect on prognosis of simvastatin in survivors of myocardial infarction: a substudy of the Scandinavian simvastatin survival study Circulation 2000 101 1366 1371 10736278 10.1161/01.cir.101.12.1366

[B34] Sanllehy C Casals E Rodriguez-Villar C Zambon D Ojuel J Ballesta AM Ros E Lack of interaction of apolipoprotein E phenotype with the lipoprotein response to lovastatin or gemfibrozil in patients with primary hypercholesterolemia Metabolism 1998 47 560 565 10.1016/S0026-0495(98)90240-2 9591747

[B35] Pena R Lahoz C Mostaza JM Jimenez J Subirats E Pinto X Taboada M Lopez-Pastor A Effect of apoE genotype on the hypolipidaemic response to pravastatin in an outpatient setting J Intern Med 2002 251 518 525 10.1046/j.1365-2796.2002.00991.x 12028507

